# Moderate injury in motor-sensory cortex causes behavioral deficits accompanied by electrophysiological changes in mice adulthood

**DOI:** 10.1371/journal.pone.0171976

**Published:** 2017-02-14

**Authors:** Wei Ouyang, Qichao Yan, Yu Zhang, Zhiheng Fan

**Affiliations:** 1 College of Physical Education and Health Sciences, Zhejiang Normal University, Jinhua, Zhejiang, China; 2 Department of Physiology, Shanxi Medical University, Taiyuan, Shanxi, China; Bilkent University, TURKEY

## Abstract

Moderate traumatic brain injury (TBI) in children often happen when there’s a sudden blow to the frontal bone, end with long unconscious which can last for hours and progressive cognitive deficits. However, with regard to the influences of moderate TBI during children adulthood, injury-induced alterations of locomotive ability, long-term memory performance, and hippocampal electrophysiological firing changes have not yet been fully identified. In this study, lateral fluid percussion (LFP) method was used to fabricate moderate TBI in motor and somatosensory cortex of the 6-weeks-old mice. The motor function, learning and memory function, extracellular CA1 neural spikes were assessed during acute and subacute phase. Moreover, histopathology was performed on day post injury (DPI) 16 to evaluate the effect of TBI on tissue and cell morphological changes in cortical and hippocampal CA1 subregions. After moderate LFP injury, the 6-weeks-old mice showed severe motor deficits at the early stage in acute phase but gradually recovered later during adulthood. At the time points in acute and subacute phase after TBI, novel object recognition (NOR) ability and spatial memory functions were consistently impaired in TBI mice; hippocampal firing frequency and burst probability were hampered. Analysis of the altered burst firing shows a clear hippocampal theta rhythm drop. These electrophysiological impacts were associated with substantially lowered NOR preference as compared to the sham group during adulthood. These results suggest that moderate TBI introduced at motorsenory cortex in 6-weeks-old mice causes obvious motor and cognitive deficits during their adulthood. While the locomotive ability progressively recovers, the cognitive deficits persisted while the mice mature as adult mice. The cognitive deficits may be attributed to the general suppressing of whole neural network, which could be labeled by marked reduction of excitability in hippocampal CA1 subregion.

## Introduction

TBI is a significant global public health problem and is a common condition affecting children all over the world [1, 2]. Though Mild TBI comprises about 80% of incidence in the young population [2], the outcome of moderate and severe TBI is often devastating. Although the underlying cellular and molecular pathophysiology are not fully understood, TBI can cause long-term motor sensory dysfunction and child death [1, 2], and often resulting in lasting serious cognitive sequelae [3].

Several moderate/severe pediatric TBI studies demonstrate frontal white matter damage, including traumatic axonal injury in children with deteriorated several cognitive courses, such as processing speed, working memory and executive deficits [4–6]. Besides cortical atrophy, juvenile rats who suffered either repeat mild TBI or moderate TBI revealed marked overall loss of neurons within cortical regions, including the motor and smatosensory cortex [7, 8]. These clinical and experimental observations suggested that frontal white matter tracts were highly vulnerable to pediatric TBI, the reduced microstructural integrity of the cortical and subregion connection, may act as a neuropathological mechanism contributing to TBI induced cognitive deficits.

Moderate/severe TBI causes neuronal cell and dendritic spines loss in mouse or rat hippocampus [9–11]. However, the association of cognitive function with neural activity is not well documented. Regionally increased or decreased the synaptic efficacy and circuit excitability was seen in the animal hippocampus following moderate/severe TBI [9–11]. No significant change was reported in the overall mean firing and burst spike frequency in CA1 subregion after mild TBI [12]. Bursting is one of the two main discharges of pyramidal neurons in the hippocampal place field, and correlate with slow theta rhythm oscillations in the local field potential of the hippocampus [13, 14]. While reductions in firing frequency and bursting of CA1 and CA3 principal cells had been proposed to be one of the mechanisms underlying spatial learning deficits [15, 16], clinical data also shows that theta burst stimulation (TBS) delivered to the dorsolateral prefrontal cortex [17], or fornix [18] may improve cognitive performance [17, 18]. However, the electrophysiology data in TBI animal seems inconsistent with the clinical results. With a mild TBI model in adult rat, recording in CA1 and CA3 revealed no significant change in firing or spike characteristics of single-neuron [19], but in a similar TBI adult rat model, burst activity of single-neuron was found to be decreased [12].

Age at the time of TBI is an important factor affecting the long-term functional outcome. Brain’s sensitivity to the deleterious effects of a TBI is varied across one’s lifetime [20]. A few experimental TBI studies have evaluated and revealed prolonged cognitive deficits [21–23]. Little is known regarding the post TBI changes of cognitive function and hippocampal electrophysiological firing during an animal transition from adolescence to adulthood. An epidemiologic study shows that across all age groups, the incidence of hospitalized and fatal brain injuries consistently peaked among late adolescents [1]. Using a lateral fluid percussion (LFP) method, which is one of the most often used experimental TBI models, and can faithfully reproduce concussion injury in human [24], our study attempts to examine how moderate TBI in the motorsenory region affects the locomotion ability, cognitive memory, hippocampal burst firing in the 6-weeks-old mice in acute and subacute phases, while the mice grow up into adult mice.

## Materials and methods

### TBI model

Male C57BL/6 mice (15–20 g, 6 weeks old at the time of injury, Research Animal Center of Shanxi Medical University) were used for the experiments. Animals were randomly assigned to TBI (n = 15) or sham (n = 15) groups, but the final TBI group include fourteen mice by excluding one mouse who died after LFP. Animals were housed under controlled conditions with a 12-h light-dark cycle. The mice had free access to water and standard food. All animal handling and procedures were approved by Animal Care and Use Committee of Shanxi Medical University, and complied with the US National Institutes of Health Guide for the Care and Use of Laboratory Animals.

Moderate TBI was induced in mice using a modified LFP method [24]. Regarding the anesthetics used in surgical anesthesia, compared with pentobarbital, inhalational anesthetic such as isoflurane was reported either having the potential of improvement for post injury recovery [25], or having similar effects on TBI induced injury pattern and severity [11]. In this study, we used the sodium pentobarbital (45 mg/kg i.p.) for animal anesthesia. In brief, under anesthesia with sodium pentobarbital, animals were fixated in a stereotaxic frame.

**Fig 1:**
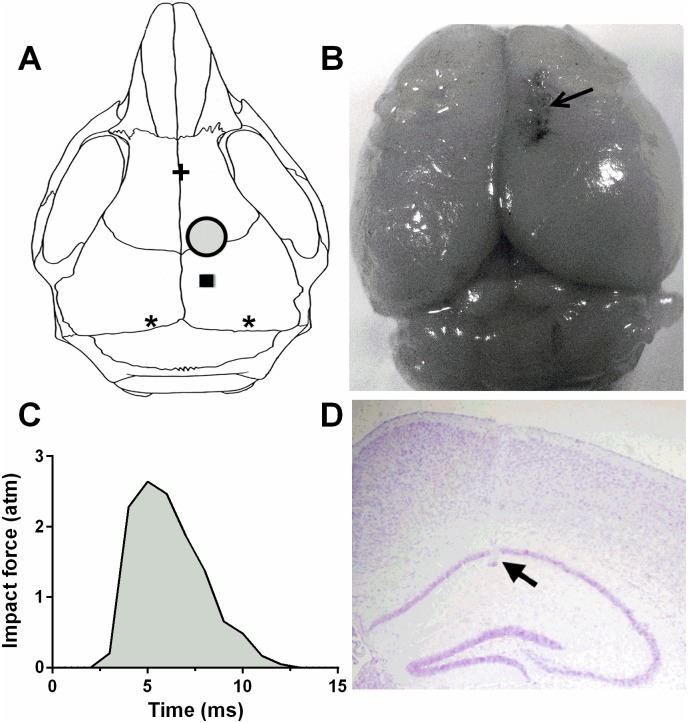
LFP, electrode implantation, injury, percussion trace and electrode location. **(A)**, Positions of LFP and recording electrodes. The large filled circle indicates the craniectomy site for LFP. The small black square shows where the recording microwires implanted. The two stars represent the screws where grounding silver leads connected with. The screw in the frontal skull, as well as the grounding screws, help to secure the whole electrode assembly. **(B)**, Macroscopic findings were seen in LFP injured mouse on DPI 16 showing the lesion confined to the cortex. **(C)**, The single LFP waveform demonstrates a rapid upstroke followed by a slower downstroke within 10 ms pulse duration. **(D)**, An example of cresyl violet staining shows the actual location of the recording electrode in mouse CA1 subregion.

Given the prevalence damage of frontal white matter including motorsensory cortex in children [4–6], also to avoid hippocampal varied condition of distortion and exposure due to later on cortical necrosis and deletion, which often occurs in the popular parietal cortical LFP animal model, we choose to LFP site a little far away from the hippocampus. A 3 mm craniectomy was performed on the right side of frontal motorsenory cortex, 1.5 mm anterior to the bregma, 1.5–2.0 mm lateral to the midline. A modified Luer-Lock (3mm inside diameter, made from a 19G needle cap) was glued to the craniectomy site and fixate with dental cement. The depth of the anesthesia was monitored by checking the palpebral and paw withdrawal reflexes. During the late period of animal anesthesia after the return of a withdrawal reflex to a paw pinch, TBI was induced using a modified fluid percussion device (Jiaxing Bocom Biotech Inc, China). A strike with the pre-adjusted pendulum generated rapid injection of saline into the closed cranial cavity which was filled with saline, thus produced a pulse of 10–12 ms duration (Fig 1A and 1C). The force of the pressure pulse was measured extracranially by a transducer and recorded by the device equipped software. The percussion force is moderate (2.5–2.8 atm) [24], which was also verified during our preliminary experiments of the cortical injury. Sham-injured animals received identical anesthesia and craniotomy, but were not exposed to LFP brain injury. Following TBI or sham injury, mice were disconnected from the device, the incision was sutured, and antibiotic ointment was applied to the wounds, the animals were kept onto the blanket which was maintained at 37°C until recovery of spontaneous motor activity.

### Measure hypnosis time

After moderate TBI, we compared its effect of the duration of pentobarbital-induced loss of righting reflex (hypnosis) between TBI and sham groups. The hypnosis time was measured as the time from which righting reflex was lost until it was regained. The righting reflex was deemed to be lost when an animal did not right itself within 15 s of being placed on its back [26], and righting reflex was assessed every 60 seconds after TBI until a normal response was observed.

### Electrode implantation and verification

Four to Five days after LFP, sham and injured and sham animals were re-anesthetized (sodium pentobarbital, 45 mg/kg i.p.), and transferred to a Kopf stereotaxic apparatus. According to the atlas of mouse brain [27], a one-row array of 7 Teflon coated Nichrome microwire electrodes (first microwire serve as the reference, 50 μm diameter, 250 μm inter-electrode distance, Biographics) was guided into the hippocampus CA1 subregion ipsilateral to the LFP side. The stereotaxic coordinates were as following, 2.0 mm posterior to the bregma, 2.0 mm lateral to the midline, 1.1–1.3 mm ventral to the skull surface. Ground electrodes were placed on the skull near the Lambda suture. A small amount of a mixture of mineral oil and bone wax was packed around all the electrode penetration zones. One support screw was placed over the frontal part of the skull, and the whole ensemble was secured with dental cement (Fig 1A).

At the completion of all experiments, the final electrodes position was marked by 20 s of stimulation at 15 μA, mice were deeply anesthetized and intracardially perfused with ice-cold sodium phosphate buffer saline followed by 4% paraformaldehyde (PFA). Brains were then removed and post-fixed in PFA for at least 24 h. After hydrate and clear sections in alcohol and xylene respectively with 2 or 3 changes, paraffin-embedded sections of brain tissue (8 μm thick) were stained with cresyl violet for microscopic examination of electrode placements (Fig 1D). Only evidence obtained from electrodes confirmed to be within the target region were used in further analysis.

### Motor performance

Motor function was evaluated with the well-established beam-walk task, and modified from the model developed by Luong and his colleagues [28]. The beam device consists of 1-meter long beam with a flat surface of 6 mm width resting 1.5 m high on two poles. The beam-walk test consisted of training and assessing the time of mice walking across the beam to a darkened goal box (30x18x22 cm), and reinforced with adverse bright light at the start end. The time of animals to cross the center 80 cm is measured were automatically measured by two infrared sensors. The mice were trained for five days with three trials per day until the animal could run the length of the beam with no more than two-foot slips. The baseline performance was recorded on the day prior surgery. The test was conducted on DPI 1, 3, 7, 9, 11, 14 and 16, and consisted of three trials with 60 sec inter-trial interval. A slip is defined as the foot coming off the top of the beam. Total hind limb steps/foot slips were manually counted, and all training and testing procedure were videotaped for later finer analysis of slipping and other observable motor deficits.

### Novel object recognition test

On DPI 7 and 14, animals were assessed NOR test, which is grounded on ‘spontaneous novelty preference’. Mice were allowed to freely explore the open field (40×40×40 cm) for 5 min, 24 hours before the test. No objects were placed inside the box during the habituation phase. During the acquisition phase, two identical objects (A and B), were placed in a symmetric position within the field. The shapes of objects are like square building blocks which are 5x5x5 cm approximately. Each animal was then placed inside the box and allowed to explore the objects for 5 minutes, and the exploration was video tracked with a camera. Furthermore, the times spent on exploring each object during acquisition and testing phase was manually recorded, and verified using the captured video. Exploration of an object was defined as rearing on it or sniffing it at a distance of less than 2 cm and/or touching it with the nose. If the mouse stands on the object or grooms within 2 cm to the object, the results were excluded. Between trials, the objects were washed with 70% ethanol solution to minimize olfactory cues. On NOR test phase (DPI 9 and 16), which is 24 hr after acquisition phase, all the processes are the same as those in acquisition phase except one of the old objects is randomly replaced with a new different object (C), a round building block (5cm diameter x 6cm high). The preference rates are measured by dividing the total exploration time by the time to explore an object. The formula to calculate the object preference is: the preference % = time to explore the individual object / total exploration time to objects x 100% [29]. The one-way ANOVA is used for statistical analysis.

### Morris water maze test

Spatial memory performance was evaluated in the Morris water maze (MWM) acutely (acquisition trials on DPI1–5 and probe trial on DPI 6), subacutely (acquisition trials on DPI 8–12 and probe trial on DPI 13). The apparatus (Zhenghua Bio Instruments Ltd., Huaibei, China,) consisted of a circular water tank (120 cm in diameter and 50 cm high) filled with water to 29 cm depth with several highly visible cues located on the inner walls of each of the four quadrants and the water temperature was maintained 23–25°C. To improve the contrast to the black color of the C57BL/6 mice, the water of the pool was made opaque using milk. Mice had to locate an invisible platform which was positioned at the midpoint of the target quadrant, and submerged 1 cm below the water level. Data were monitored and achieved using a video camera with a computerized video tracking system (Ethovision 3.0, Noldus Information Technology, Wageningen, the Netherlands). Each animal was trained four times per day for 5 consecutive days. For each trial, mice were randomized to one of four quadrants and placed in the pool facing the wall. Mice were given a maximum of 60 seconds to find the submerged platform. Mice who successively found the platform was allowed to rest on it for 10 sec, while mice who failed to locate the platform within the time limit were guided to it and allowed to rest for 20 sec. On day 6, all mice also underwent a probe trial (retention phase), where the platform was removed from the pool. Performance in the MWM was quantitated by latency to find the platform, and the total time spent in the target quadrant (where the platform had been removed) versus the other quadrants.

### Electrophysiological recording and analysis

Electrophysiological recording was carried out while mice freely explore the open field on DPI 7 and 14. Since no “place cell” were recognized in a similar TBI animal model during NOR test [12], and analyzing large quantity of cells is required to correctly identify “place cell” by assessing the correlation between firings rate and animal’s spatial location [30], we haven’t attempted to classify the “place cell” during NOR test. Burst firing has stronger “downstream” effects than that of a single spike, including a summation of EPSP and synaptic plasticity. Bursting of hippocampal principal cells has been implicated in spatial learning deficits [15, 16]. Burst firing was assessed in this study while animals explored the open field, 24 hr before assessing NOR. Through a microwire array connected samtec adaptor and light-weight cable, the spiking signal was digitized at 30 kHz using a 64-channel data acquisition system (Cerebus, Blackrock Microsystems) with the band-pass filtering of 250–7500 Hz with a threshold set at 4 s.d. of the background noise.

**Fig 2:**
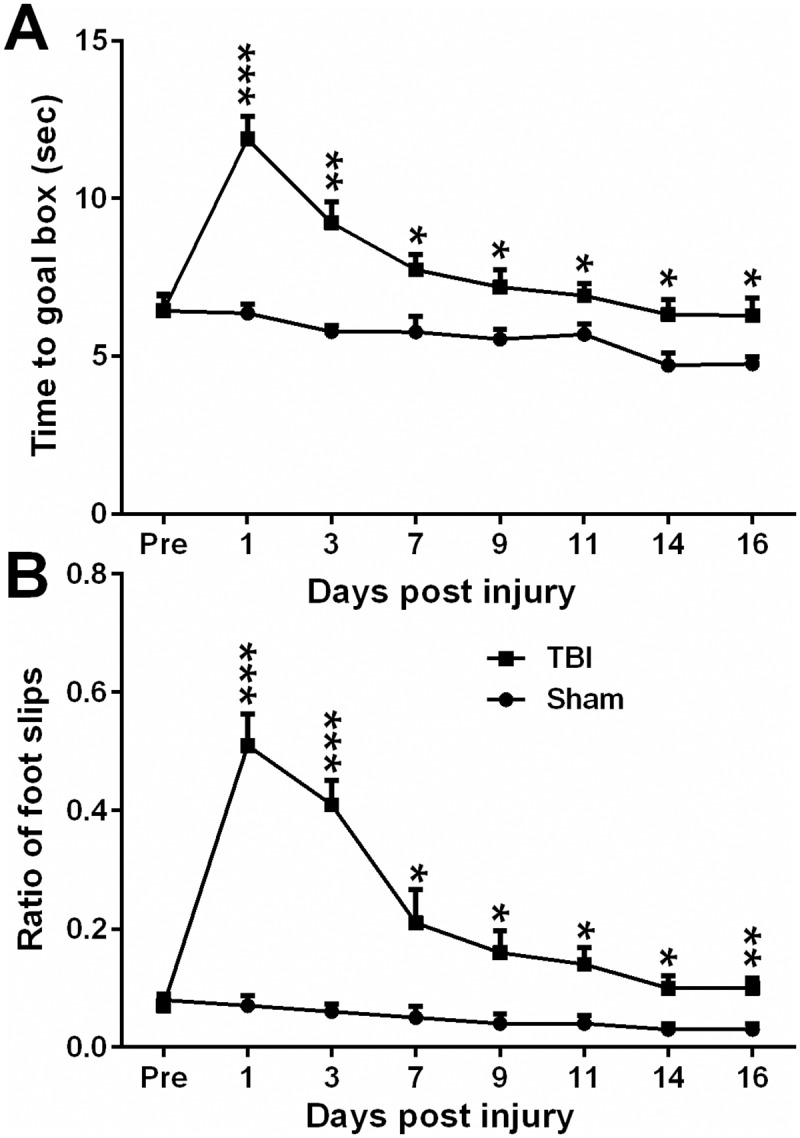
A typical burst firing recording and its inter-spike interval histogram. (A), typically isolated burst firing waveform recorded from CA1 pyramidal cell. (B), inter-spike interval histogram of the isolated burst firing in CA1 cell.

CA1 pyramidal neuron spike discharges were recognized by means of the waveform (Fig 2), spontaneous rate and distinctive phasic bursts as described previously [31]. Before including neural signals in a data set, autocorrelograms were performed on each channel record to assure those single neurons were separated from each other and from the background noise. Neural spikes of identified principal cells with firing rates of 0.25–5.0 Hz were isolated using Offline Sorter (Plexon Inc., Dallas, TX) and confirmed using NeuroExplorer (Nex Technologies, Littleton, MA). Low-frequency firing neurons of 0.25 Hz or less were excluded from the study’s analyses due to the insufficient number of spikes. Legendy and Salcman [32] assumed that the frequency of spike trains had a Poisson distribution, and defined bursts of spikes by the value of the ‘Poisson surprise’ parameter. A burst is defined as at least three consecutive spikes with two sequential interspike intervals (ISI) less than one-half of the mean ISI of all spikes. In this study, bursting feature of spikes were characterized using NeuroExplorer included bursting analysis function, which was based on ‘Poisson surprise’ method. Bursts with Surprise values > 10 were used to analyze the burst per minute, mean burst duration, mean spikes in burst, mean frequency in burst, mean ISI in burst and mean inter-burst interval (IBI).

### Histological

Once the behavioral assessment tests have been done on DPI 16, the mature adult mice were pentobarbital anesthetized, transcardially perfused with isotonic saline followed by 4% paraformaldehyde in phosphate-buffered saline (PBS, pH 7.2). Brains were removed, post-fixed in 4% paraformaldehyde for 24 h at 4°C, and sectioned coronally from 1 mm anterior to the lesion site to the posterior margin of the lesion. Then, the brain tissues were dehydrated by immersion in a series of aqueous alcohol solutions gradually moving to pure alcohol. After clearing with several xylene washes, the tissues finally were paraffin-embedded (Sigma-Aldrich). Using a microtome (Thermo Shandon Finesse ME+, Suzhou, China), the brain tissue was cut into 6 μm-thick coronal sections (300 μm apart). The sections were Hematoxylin and eosin (HE) stained using modified methods (0.5% hematoxylin for 8 min at 25°C; 0.1% eosin for 2 min at 25°C). Images were acquired using an OLYMPUS IX71 microscope (Olympus, Tokyo, Japan), and cortical lesion volumes were analyzed using NIH ImageJ software (National Institutes of Health, Bethesda, MD, USA). The area of the lesion was determined by subtracting the intact area of the ipsilateral hemisphere from the area of the contralateral hemisphere. Ten sections at 300 μm intervals throughout the entire lesion and the lesion volume was presented as a volume percentage of the lesion compared with the contralateral hemisphere. Starting at coordinates AP −1.58 mm and ending AP −3.28 mm from bregma [27], 6 μm-thick coronal brain sections with 100 μm apart covering the whole dorsal hippocampus. A total number of 5 sections per mouse were used. Cells presenting with nuclear and cytoplasmic staining (HE) were manually counted in the hippocampal CA1 neurons.

### Statistical analysis

Student t-test, analysis of variance (ANOVA) were used for group comparison, as appropriate, with Newman-Keuls or Tukey post hoc comparisons. p<0.05 was considered statistically significant. Data are presented as means ± SEM. The strength of a linear association between two group variables was determined using Pearson correlation coefficient.

## Results

### Injury

The injury severity in TBI animal is gauged by the biomechanical forces and the acute suppression of neurological reflexes. After moderate LFP strike, righting reflex was regain late in TBI group mice compare with that in sham group mice, consequently, the hypnosis duration of pentobarbital was prolonged (94±4 min, n = 7) for TBI group mice, compare with sham group mice (71±2 min, n = 8).

### Motor function

**Fig 3:**
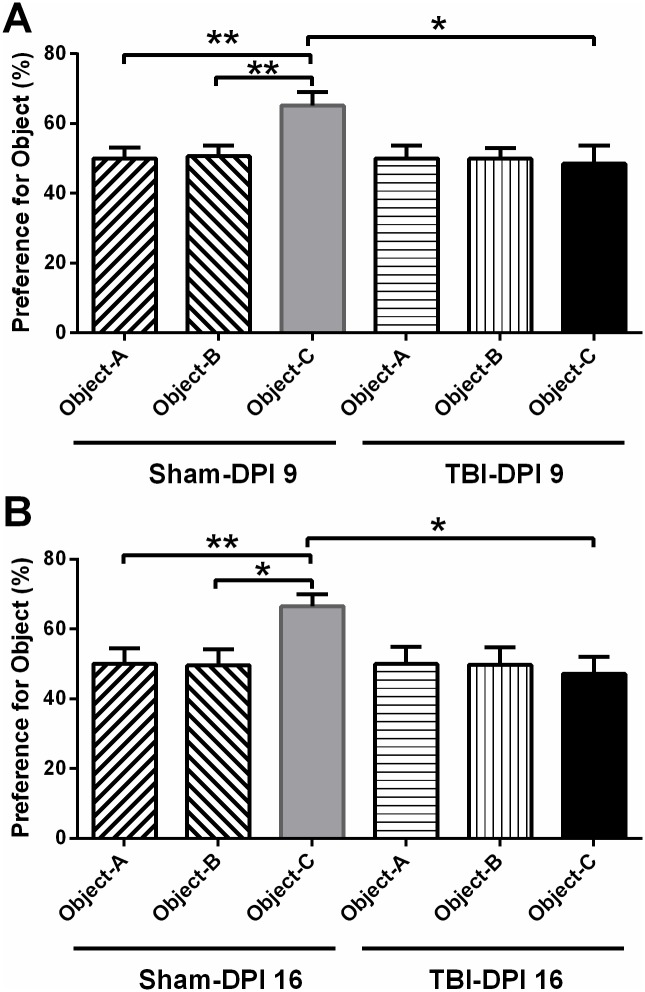
The Motor function changes after TBI. Mice motor performance in term of beam-walk latency (A) and foot-fault (B) were significantly impaired due to moderate TBI. Multiple Holm-Sidak *t* test were used to compare the daily based beam-walk or foot-fault between TBI and sham groups. Data are expressed as mean±SEM (n = 7). * p<0.05, ** p<0.01 and ***, P < 0.001 vs. control.

Beam-walk on a narrow beam (6 mm) was evaluated since mice with TBI at motor-sensory cortex lesions intend to show movement dysfunction of the contralateral side of the body. The narrow beam was chosen because C57BL/6 mice are typically active rodent animals [33]. No pre-surgical differences in time to traverse the beam were revealed between TBI and sham groups, as all mice were proficient and reached the goal box in approximately 6–7 sec. After TBI, two-way ANOVA analysis of beam-walk data shown significant group (F1,80 = 83.52, p < 0.0001) and day (F7,80 = 12.75, p< 0.0001) differences vs. sham controls; similarly two-way ANOVA analysis of foot-fault data also confirmed significant group (F1,80 = 135.5, p < 0.0001) and day (F 7,80 = 18.55, p < 0.0001) differences vs. sham controls. Exact daily comparison of beam-walk and foot-fault function between TBI and sham groups was conducted using multiple *t* tests, and results are shown in Fig 3. In addition, the beam-walk deficits significantly correlated with the foot-fault performance from DPI 1 to 16 (Pearson correlation r = 0.9749; p = 0.000937).

### Novel object recognition

**Fig 4:**
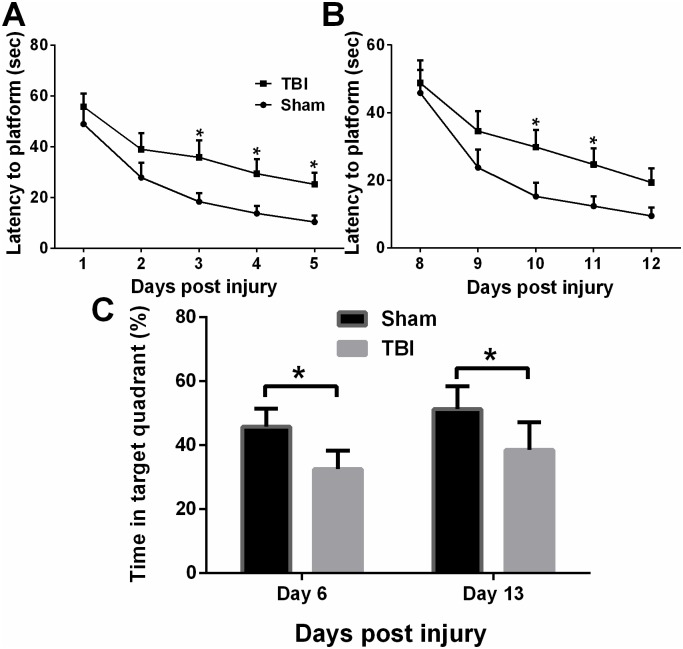
Object preference (mean percent exploration time) evaluation on DPI 9 and 16. The sham group performs significantly better than TBI group on the novel object (object-C) testing on both DPI 9 (**A**) and 16 (**B**), indicating TBI consistently deteriorated cognitive memory after injury. Object-A, B, identical objects; Separate ANOVAs were used to compare the preference for all the familiar and novel objects during the acquisition phase (object-A, B test) and following novel object test phase (object-A/B and object-C test). Data are expressed as mean±SEM (n = 7–8). * p<0.05, ** p<0.01 vs. control.

During the acquisition phase, the exploration times for two identical objects are similar (around 50%) in TBI and sham group mice. During NOR test phase on both DPI 9 and 16, sham group mice spent significantly more time with higher preference rates on the novel object demonstrating good NOR performance, while TBI group animal explored significantly less than sham groups indicating damaged NOR performance (Fig 4). Thus, TBI injury resulted in significant and consistent object memory impairment in TBI injured mice during subacute phase while mice transit from late adolescence to young adult. Moreover, the injury severity as measured by the prolonged loss of righting reflex after TBI significantly correlated with the worse NOR performance on DPI 9 (Pearson correlation r = 0.778; p = 0.039), and on DPI 16 (Pearson correlation r = 0.769; p = 0.043), as measured by the preference to the novel object.

### Spatial memory

**Fig 5:**
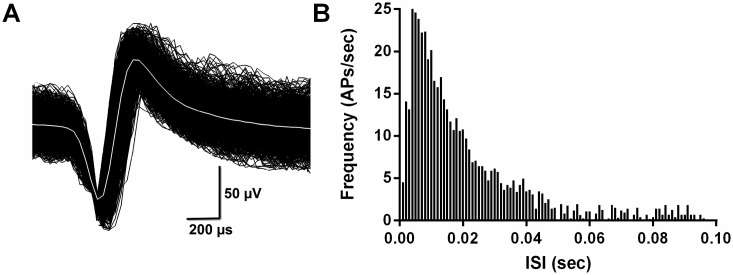
TBI impaired learning and spatial memory retention in mice. TBI mice demonstrated an increased latency to find the platform, compared to the sham mice in acquisition phase (**A**, DPI 1 to 5 and **B**, DPI 8 to 12). During trials in probe phase (DPI 6, p<0.05 and DPI 13, p<0.05), TBI mice spent a significantly less percentage of time in the target quadrant, compare to the sham mice. Data are expressed as mean±SEM (n = 7). * p<0.05, vs. control by control by Multiple Holm-Sidak t test or ANOVA with Newman-Keuls *post hoc* test.

Besides the long-term visual memory evaluation in NOR test, we also assessed spatial learning and long-term memory in the moderate TBI mice using the MWM. During the acquisition phase, mice from both groups showed daily progress in their abilities to locate the hidden platform. However, moderate TBI mice displayed increased latencies versus the sham group mice (Fig 5). At the acute time points (DPI 1 to 5), two-way ANOVA analysis showed that the group (F1,50 = 16.23, P < 0.001) and day (F4,50 = 13.83, P < 0.0001) differences were statistically significant vs. sham group (Fig 5A). The group (F 1,50 = 10.14, P < 0.01) and day (F4,50 = 13.21, P < 0.0001) difference were also significant at subacute time points (DPI 8–12, Fig 5B). Subsequent analysis was performed to evaluate the preference for the target quadrant between sham and TBI group. The mice in the sham group spent significantly more time in the target quadrant than the mice in TBI group on the probe trial days (DPI 6 and DPI 13, Fig 5C).

### Bursting changes in CA1

Applying the criteria and identify approaches mentioned in the method, 24 units were identified in sham and TBI group respectively on DPI 7, while 23 and 24 units were recognized in sham and TBI group respectively on DPI 14. A representative isolated burst firing waveform recorded from CA1 pyramidal cell, and its related inter-spike interval histogram are shown in Fig 2. Compare with sham group, burst firing pattern of CA1 pyramidal cell was altered while TBI mice moving freely in the open field, showing a trend of more unpredictable, which was denoted by significantly increased burst surprise values on both DPI 7 (12.52 ± 0.45 and 19.82 ± 1.33 for sham and TBI group respectively, n = 24, p<0.001 vs. sham by unpaired *t*-test.) and 14 (12.72 ± 0.46 and 25.58 ± 2.23 for sham and TBI group respectively, n = 23–24, p<0.001 vs. sham by unpaired *t*-test.).

**Fig 6:**
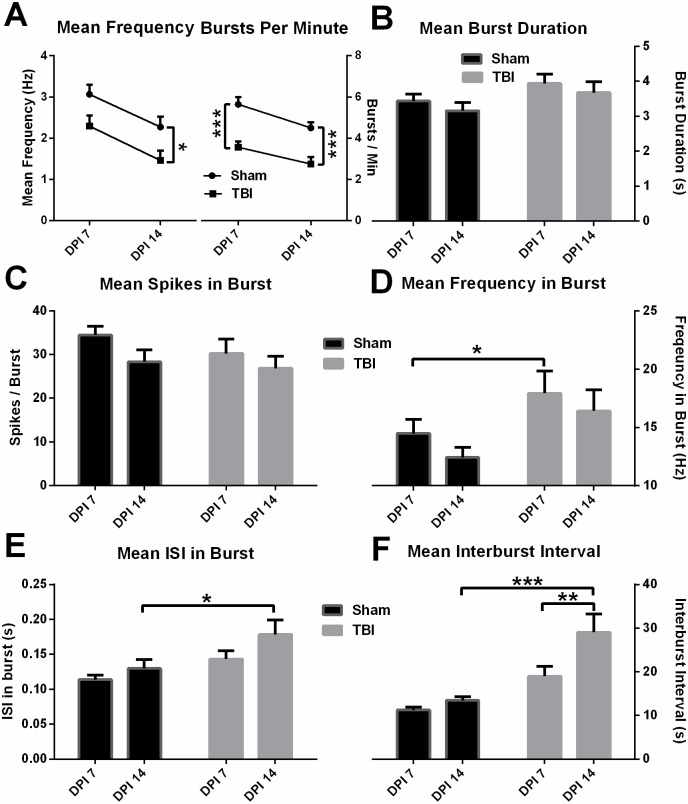
Several aspects of burst firing in CA1 were altered following moderate TBI. Significant decrease of overall mean frequency on DPI 14 (**A left inset**), and burst per minute on both DPI 7 and 14 (**A right inset**), increase of burst duration on DPI 14 (**B**), reduction of mean spikes in burst (**C**), significant increase of mean frequency in burst on 7 DPI (**D**), significant increases of mean ISI in burst (**E**) and mean interburst interval (**F**) on DPI 14. Data are expressed as mean±SEM (n = 23–24). * p<0.05, ** p<0.01, *** p<0.001 vs. control by unpaired *t*-test or ANOVA with Newman-Keuls *post hoc* test.

**Fig 7:**
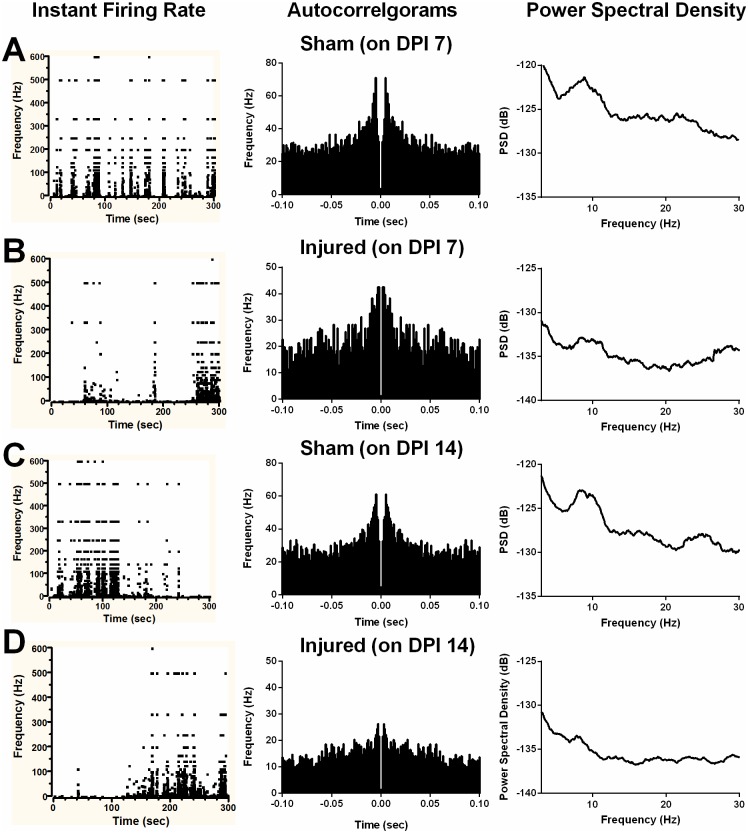
Results of burst analysis in the CA1 subregion after moderate TBI. Representative instant firing, autocorrelogram and power spectral density in CA1 pyramidal cell of sham mice on DPI 7 (**panel A**) and 14 (**panel C**). Decreases of instant burst firing (**left column**) in CA1 pyramidal cell of TBI mice on DPI 7 (**panel B**) and on DPI 14 (**panel D**) are confirmed by the reductions of frequency in autocorrelogram (1ms bin, **middle column**) and drops of power spectral density within theta range (**right column**). Data in three graphs of each panel (A-**D**) are from the same cell.

Moderate TBI significantly impaired the overall firing rate in TBI mice on DPI 14 (Fig 6A left), while significantly suppressed burst per minute in TBI mice on both DPI 7 and 14 (Fig 6A right). In the meantime, the mean burst duration presented a trend of an increase (Fig 6B), while the mean spikes in the burst (Fig 6C) showed a trend of decrease though both changes were not significant. On the contrary, mean frequency in burst (Fig 6D), mean ISI in burst (Fig 6E) and mean interburst interval (Fig 6F) was significantly increased in TBI group during adulthood on DPI 7 and/or 14. Accordingly, parallel with the increased bursty feature, above electrophysiological data demonstrate that CA1 cells in TBI group progressively fire fewer bursts of spikes with longer intraburst and interburst interval. However, the TBI affected CA1 cell showed faster firing within the burst. Representative instant burst firings (Fig 7 left column), firing probability (Fig 7 middle column) and power spectral analysis (Fig 7 right column) are shown in Fig 7. The instant bursting firing rate was hampered in TBI mice at both acute (DPI 7, Fig 7C) and subacute (DPI 14, Fig 7D) phase vs. control (DPI 7, Fig 7A; DPI 14, Fig 7B). Moreover, the burst firing probability drops were also defined in autocorrelogram (Fig 7 middle). Using neuroexplorer, the power spectral analysis of neural firing histograms revealed that both sham and TBI groups’ firing show a clear hippocampal theta rhythm drop (4–12Hz), and the spectral theta band energy substantially declined in TBI groups (Fig 7 right).

### Histological

Sham animals had no volume loss. After pediatric TBI, there was an evident tissue loss with numerous neuronal death at adulthood at the LFP region in the ipsilateral hemisphere (S1A–S1D Fig). The cortical tissue loss in TBI group, represented as the volume percentage of the lesion compared with the contralateral hemisphere is 3.01 ± 0.64% (Mean ± SEM, n = 7).

Where dorsal hippocampal CA1 subregion is proposed to be involved in memory and recognition functions [34], we focus on the neural appearance in CA1 layer area. After moderate TBI, the morphological alterations demonstrated by HE staining around pyramidal cell layer in the ipsilateral CA1 were shown in S1E and S1F Fig. Moderate TBI didn’t cause significant neural degeneration. The percent of shrunken cells with nucleus pyknosis in CA1 were 3 ± 1% in the sham group, 6 ± 1% in TBI group (Mean ± SEM, n = 7–8, p = 0.06 by one tailed *t* test). However, adverse cell morphological changes, such as irregular morphology, fading contour and slightly swelled were seen in the ipsilateral CA1 pyramidal cell layer (S1E and S1F Fig).

## Discussion

The primary damage of moderate/severe TBI can occur in specific brain areas; motor and somatosensory cortices are among the most vulnerable targets in patient suffered moderate/severe TBI with diffuse axonal injury. Secondary injury can dissipate progressively throughout the brain [35]. The layered fundamental structure of the mouse cerebral cortex, layers 1, 2/3, 4, 5, and 6 are named by homology with other mammals, and have similar cell types in each layer [36]. Further, the functions in frontal are similar between mouse and human, e.g. somatosensory cortex in human also contribute to motor control, and similarly the mouse barrel field controls retraction of the whiskers [37]. While there is currently a lot of ongoing researches on the acute (DPI 1–6) phase of TBI, much less research has been done on the subacute (DPI 7–30) and the chronic (DPI 31–90) phases. Using a moderate (2.5–2.8 atm) LFP model of brain injury in the 6-weeks-old mice, we have demonstrated that TBI at the motorsensory region is associated with impaired motor and cognitive functions, decreased overall firing and bursting probability in hippocampal CA1 subregion at both acute and subacute phases, where the mice become adult mice. In addition, moderate TBI causes evident tissue loss and neural cell death in the ipsilateral motor cortex, and causes cell stress, at large scale in hippocampal CA1 subregion.

After TBI, the motor deficits were the worst on DPI 1 and gradually improved from DPI 1 to 16, the subacute and adulthood phase. Similar daily difference between beam-walk latency (F7,80 = 12.75) and foot-fault (F7,80 = 18.55) from DPI 1 to 16. Moreover, the two deficits recovery processes showed a close correlation (r = 0.9749), and tend to be stabilized from DPI 14 to 16. However, these deficits still maintain significant difference compare with the control. The recovery of motor deficit transition in present TBI mouse model coincides with the previous findings that the locomotor ability of moderately parietal cortex injured mice [38], even motor cortex ablated rats [39] mostly recovered within 3–4 weeks. The initial loss of motor function and subsequent recovery may be the manifestation of a compensatory transition of motor control to some intact areas.

24 hr after the acquisition phase, LFP injured mice showed similar poor performance during NOR test at two subacute time points (DPI 9 and 16). Additionally, the poor NOR performance in TBI mice is significantly correlated with their injury severity. Unlike the motor deficits, the NOR performances appear to worsen similarly on DPI 9 or 16. Furthermore, there was a close correlation between NOR performance and the initial injury severity (r = 0.778 on DPI 9, r = 0.769 on DPI 16) which was indicated by the loss of righting reflex after TBI. Therefore, the basic locomotion ability was largely recovered, while the visual-based memory deficit is more established in the subacute period, while the 6-weeks-old mice grow up.

Our observation on NOR agreed with previously published findings. When long-term memory performance was also tested by defining the NOR 24 hr after the acquisition trial, controlled cortical impact (CCI) injury at moderate degree induces severe NOR deficits in adolescent rat during adulthood at three weeks [21, 22] and six weeks [21] post injury. Further, the worse long-term NOR performance at three weeks did not differ with respect to the performance at six weeks [21]. However, the NOR performance was varied in a weight drop TBI model similarly at moderate degree, injured mice showed better NOR performance on DPI 28 compared to the performance on DPI 3 [23].

We also evaluated the long-term spatial learning and memory change using Morris water maze after moderate TBI. The long-term spatial memory was damaged in acute phase in young adult TBI mice, and consistently damaged in subacute phase when young mice grew up into adult mice.

Previous investigations of the neural basis of recognition memory have implicated several brain regions, including perirhinal cortex, medial prefrontal cortex, hippocampus and medial dorsal thalamus [40]. Structures in the medial temporal lobe, including the hippocampus and perirhinal cortex, are known to be essential to the formation of long-term memory. Stimulation of perirhinal cortex at specific frequencies can predictably modify recognition memory [41]. The functional interaction between the hippocampus and medial prefrontal cortex (PFC) is also important for executive function and working and long-term recognition memory [42, 43]. In humans and non-human primate’s injury to the medial dorsal thalamus also yields recognition memory deficits [44, 45]. The consistent findings of long-term memory deficit above mentioned indicate that moderate TBI may cause damage to long-term neural circuitry.

Hippocampus is critical during cognitive development in children from procedure-based to memory-based problem-solving strategies; parallels increased hippocampal-neocortical functional connectivity, increased hippocampal and decreased prefrontal-parietal engagement [46]. However, the exact role of the hippocampus within recognition memory neural circuit has not been fully elucidated. The patient data also deliver a contradictory conclusion on the contribution of hippocampal volume reduction in cognitive deficits [47–50], and there is insufficient literature about electrophysiological aberrations that may add to the TBI induced cognitive dysfunction.

In the present study, we demonstrated that moderate TBI is associated with firing significant decrease of overall firing and bursting probability in hippocampal CA1 subregion during adulthood. After TBI, the burst firing pattern is less organized with longer burst duration, higher frequency in burst, wider ISI in burst, broader interburst interval in the hippocampal CA1 subregion. The burst probability was dropped in TBI group mice, defined with significantly increased burst surprise values. In similar TBI rat models, TBI cause massive increases in extracellular potassium ion ([K^+^]_e_) [51, 52], induce long-lasting (1 to 30 days after TBI) elevations in intracellular free calcium ([Ca^2+^]_i_)[53–55] in hippocampus, and this increase of [K^+^]_e_ may be related to the indiscriminate release of glutamate following TBI [51]. In addition, the mild TBI induced increase of [K^+^]_e_ could be blocked by tetrodotoxin, suggesting that this rise in [K^+^]_e_ is related to neuronal firing. These two critical ion concentration changes might alter the transmembrane ion equilibrium, e.g. hyperpolarizing outflow of K^+^ could be slowed down due to a reduced K^+^ gradient, and the membrane potential could subsequently be changed. These TBI induced abnormal ion distribution might be the neurochemical signs for neural cell firing changes, and there should be a reciprocal relationship between these abnormal neurochemical and neural cell firing changes. We postulate that the re-establishing process of the ionic equilibrium after neuronal firing may be longer in TBI injured hippocampus. Therefore, the bilateral interaction mentioned above may contribute to the altered burst firing pattern, as we found in hippocampal CA1 of TBI mice, e.g. increased mean frequency in burst, wider ISI in burst and broader interburst interval. Furthermore, these long-term alterations in Ca^2+^ dynamics were associated with the TBI impaired cognitive function [55]; administration of VGCC antagonists could reduce cell death and improved cognitive function [56].

The hippocampal theta rhythm is a well-studied correlate of memory function. Theta power and peak theta frequency were reported significantly attenuated in the LFP injured animals [57]. Burst firing in the hippocampus was stated locking a preferred phase of either main slow oscillations or theta rhythms within the local field potential [58]. Theta burst stimulation delivered to the fornix of mTBI rats improved learning and memory [59], similarly, theta burst stimulation supplied to the medial septal nucleus of mTBI rats improved spatial working memory [60]. Changes in theta activity in this study were evaluated by analyzing of neural firing histograms in the hippocampus while mice exploring the open field. The results demonstrated that moderate TBI is associated with steadily attenuated theta band (4–12Hz) firing at both subacute time points (DPI 7 and 14) during adulthood. In summary, the TBI deteriorated neural cell firing may further destabilize the transmembrane ionic equilibrium, and the disturbed key ion levels and Ca^2+^ homeostasis may partially bridge the altered neuronal firing and cognitive deficits. Thus specifically patterned stimulation of one or multiple targets in the memory related neural circuitry may provide a novel approach to the treatment of memory loss and cognitive deficits after TBI.

Our results of moderate TBI in mice also agree with a previous finding of mild traumatic brain injury (mTBI) in adult rats. In that research, the author has proven that mTBI (1.4–1.6 atm) is associated with significantly less hippocampal bursting with longer bursts and lower interburst spike frequency [12]. Bursts with shorter intervals in hippocampal CA1 are identified to provoke LTP [61] and have a crucial role in synaptic plasticity [62], thus lower burst firing with wider ISI in the burst and broader interburst interval suggested TBI retarded information coding in the hippocampus. Further, TBI may selectively disrupt circuit excitability in hippocampal subregions. In a hippocampal slice with moderate TBI, decreased net synaptic efficacy and excitatory postsynaptic potential-spike relationship [9] were also shown in CA1 area, while increased net synaptic efficacy [9] or hyperexcitability [11] were revealed in the dentate gyrus. With similar recording approach in moderate TBI brain slice, a decrease of excitatory synaptic inputs was also reported in granule cells [10], while reduced compound action potential amplitudes were found in the corpus callosum [8], suggesting a reduction of excitability at the global level. In a mild LFP injured rat model, the mean and burst firing was reported no change in CA1 subregion [12].

It should be noted that the TBI fabrication site is different between our study at motorsensory cortex and popular TBI investigations usually at the parietal cortex [12, 21, 38]. Histological findings in present study showed generally an evident tissue loss at LFP site in the ipsilateral motor cortex, but failed to show a significant cell degeneration in hippocampal CA1 subregion. It should be mentioned that TBI induces injury by compression of the cortex and underling brain tissues and does not directly causes hippocampal damage. The LFP site for this study is at motorsenory cortex and is a little far away from the hippocampus than the LFP location in other parietal cortex models [12, 21, 38]. Consequently, mouse hippocampus is more intact and fewer deformations though moderate TBI was introduced in the model, thus the exact injury severity and cell degeneration in the hippocampus may be primarily due to secondary but chronic brain injury, e.g. neuroinflammation [38] induced necrosis. In present study, moderate TBI causes neural cell stress but does not obviously change the architecture of the hippocampus, and the majority of the mature pyramidal neurons in the CA1 subregion survived but stressed at this level of TBI injury.

In summary, experimental moderate TBI by means of LFP in young adult mouse causes marked motor dysfunction in the acute phase after TBI, and the motor function gradually recovered in the subacute phase where the mice gradually mature as an adult mouse. Moderate TBI consistently caused long-term memory deficits, steadily decreased firing frequency, burst probability in hippocampal CA1 in both acute and subacute phases during adulthood. Depressed of overall firing and altered bursting properties in CA1 subregion may further deteriorate the neuronal ionic equilibriums, disturb the long-term memory related neural circuit at the whole hippocampal level. The adverse outcomes could be confirmed with the damaged NOR and spatial performance seen in the same animals during adulthood. Longer follow-up studies should be carried out after young adult LFP injury to inspect the hippocampal and/or cortical electrophysiological changes, the temporal evolution of memory deficits, and whether the altered firing profile progressively correlates with the memory deficits.

## Supporting information

S1 Fig HE staining of the ipsilateral motor cortex and hippocampus from sham (A, C, E) and TBI group (B, D, F) on DPI 16. The morphology of motor cortex (A) and neural cells (C) in the sham group appear normal. HE staining shows an evident tissue loss in the ipsilateral motor cortex (B, D), and numerous neural cell death in the adjacent region of TBI (D). Pyramidal cells in CA1 layer in sham group were contour-clear, arranged regularly with normal nuclei and cytoplasm (A). Neural cells in TBI group arranged disorderly, with unclear nuclear structure, and a large number of cells slightly swelled (B). As black arrows denoted, a few of focal nuclei pyknosis and/or eosinophilic neurons with H–E stain were observed in sham or TBI group. Scale bar, 100μm; magnification 40 x in A and B; scale bar, 50μm; magnification 100 x in C and D; Scale bar, 100μm; magnification 400 x in E and F.
(TIF)
