# Supplementary figures and images for: Moderate injury in motor-sensory cortex causes behavioral deficits accompanied by electrophysiological changes in mice adulthood
Source: PLoS One. 2017 Feb 14;12(2):e0171976. doi: 10.1371/journal.pone.0171976 (PMC5308857; doi:10.1371/journal.pone.0171976)

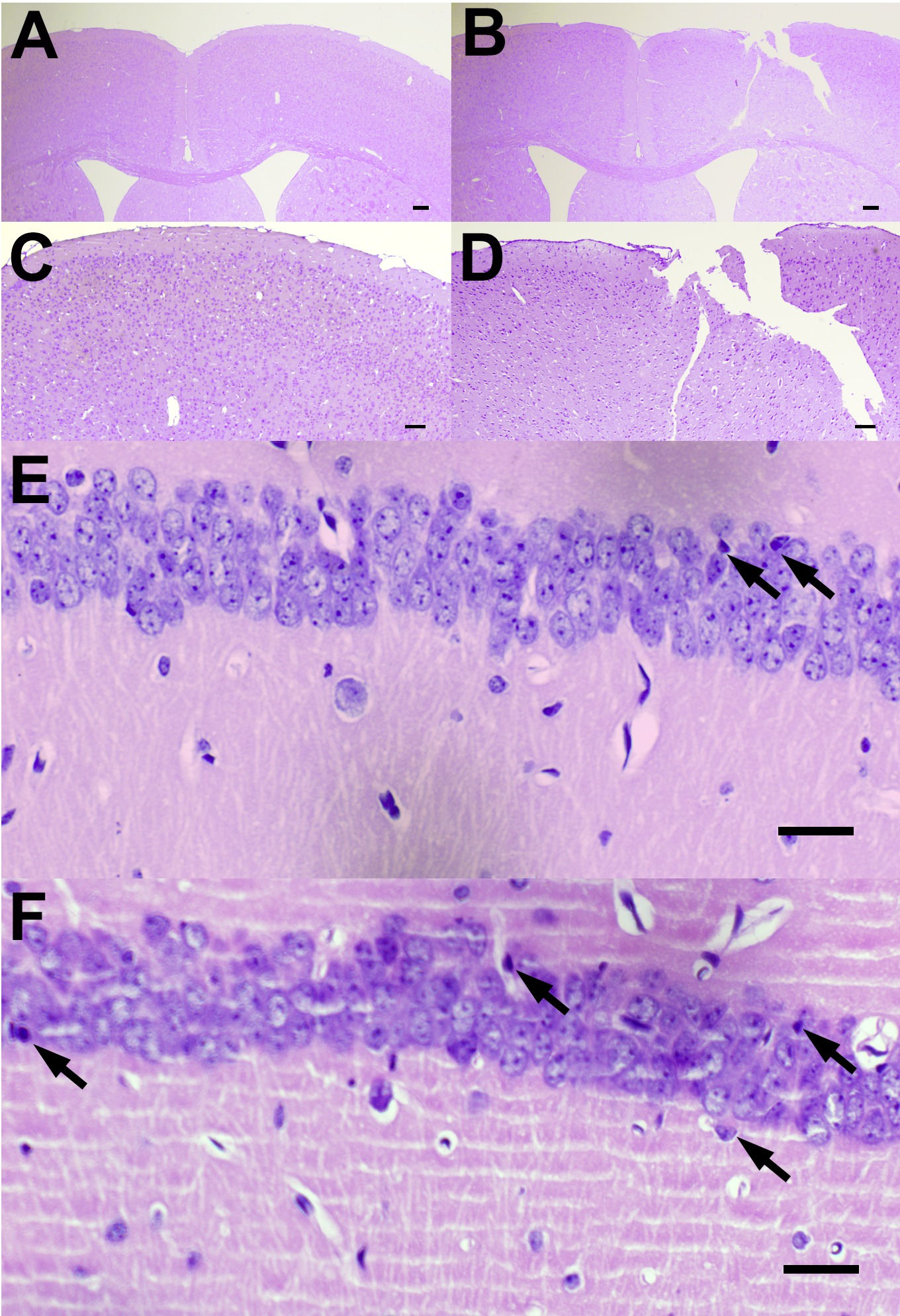

Supplement: S1 Fig — The morphology of motor cortex (A) and neural cells (C) in the sham group appear normal. HE staining shows an evident tissue loss in the ipsilateral motor cortex (B, D), and numerous neural cell death in the adjacent region of TBI (D). Pyramidal cells in CA1 layer in sham group were contour-clear, arranged regularly with normal nuclei and cytoplasm (A). Neural cells in TBI group arranged disorderly, with unclear nuclear structure, and a large number of cells slightly swelled (B). As black arrows denoted, a few of focal nuclei pyknosis and/or eosinophilic neurons with H–E stain were observed in sham or TBI group. Scale bar, 100μm; magnification 40 x in A and B; scale bar, 50μm; magnification 100 x in C and D; Scale bar, 100μm; magnification 400 x in E and F. (TIF) [file pone.0171976.s001.tif]
